# Senecavirus a 3D Interacts with NLRP3 to Induce IL-1β Production by Activating NF-κB and Ion Channel Signals

**DOI:** 10.1128/spectrum.02097-21

**Published:** 2022-03-07

**Authors:** Sk Mohiuddin Choudhury, XuSheng Ma, ZongBo Zeng, Zhikuan Luo, Yuanyuan Li, XiaoFeng Nian, YongHua Ma, Zhengwang Shi, Rui Song, ZiXiang Zhu, Weijun Cao, Jingjing Pei, HaiXue Zheng

**Affiliations:** a State Key Laboratory of Veterinary Etiological Biology, National Foot and Mouth Disease Reference Laboratory, Key Laboratory of Animal Virology of Ministry of Agriculture, Lanzhou Veterinary Research Institute, Chinese Academy of Agricultural Sciences, Lanzhou, Gansu, China; b Gansu Agricultural University, Lanzhou, Gansu, China; Regional Centre for Biotechnology

**Keywords:** Seneca valley virus A, inflammation, NLRP3, SVA 3D protein, NF-κB, ion channel

## Abstract

Senecavirus A (SVA) infection induces inflammation in animals, such as fever, diarrhea, vesicles and erosions, and even death. The inflammatory cytokine interleukin-1β (IL-1β) plays a pivotal role in inflammatory responses to combat microbes. Although SVA infection can produce inflammatory clinical symptoms, the modulation of IL-1β production by SVA infection remains unknown at present. Here, both *in vitro* and *in vivo*, SVA robustly induced IL-1β production in macrophages and pigs. Infection performed in NOD-, LRR-, and pyrin domain-containing three (NLRP3) knockdown cells indicated that NLRP3 is essential for SVA-induced IL-1β secretion. Importantly, we identified that the 1 to 154 amino acid (aa) portion of SVA 3D binds to the NLRP3 NACHT domain to activate NLRP3 inflammasome assembly and IL-1β secretion. In addition, the SVA 3D protein interacts with IKKα and IKKβ to induce NF-κB activation, which facilitates pro-IL-1β transcription. Meanwhile, 3D induces p65 nucleus entry. Moreover, SVA 3D induces calcium influx and potassium efflux, which triggers IL-1β secretion. Ion channels might be related to 3D binding with NLRP3, resulting in NLRP3-ASC complex assembly. We found that 3D protein expression induced tissue hemorrhage and swelling in the mice model. Consistently, expression of 3D in mice caused IL-1β maturation and secretion. In the natural host of pigs, we confirmed that 3D also induced IL-1β production. Our data reveal a novel mechanism underlying the activation of the NLRP3 inflammasome after SVA 3D expression, which provides clues for controlling pig’s inflammation during the SVA infection.

**IMPORTANCE** Inflammation refers to the response of the immune system to viral, bacterial, and fungal infections or other foreign particles in the body, which can involve the production of a wide array of soluble inflammatory mediators. The NLRP3 inflammasome is one of the best-characterized inflammasome leading to IL-1β production and maturation. Senecavirus A (SVA) is an oncolytic virus that can cause fever, vesicles and erosions, severe fatal diarrhea, and even the sudden death of piglets. In this study, we demonstrated that 1 to 154 aa of SVA polymerase protein 3D interacts with the NACHT domain of NLRP3 to induce IL-1β production via the NF-κB signaling pathway and ion channel signal. Our study unveils the mechanism underlying the regulation of inflammasome assembly and production of IL-1β in response to SVA infection that will help better understand the modulation of host inflammation in pathogens invasion and development of the vaccine.

## INTRODUCTION

SVA was first isolated in 2002 and was proposed for use as an oncolytic virus to cure neoplasia (1, 2). It is the only member belonging to the *Senecavirus* genus of the Piconarviridae family. SVA is a positive-sense single-stranded RNA virus that consists of structural proteins (VP1, VP2, VP3, and VP4) and non-structural proteins (L^pro^, 2A, 2B, 2C, 3A, 3B, 3C, and 3D) (3). SVA infection can cause fever, vesicles and erosions, and severe fatal diarrhea in piglets, and can lead to epidemic transient neonatal losses and the sudden death of piglets (2, 4). SVA is associated with inflammatory symptoms; however, the mechanisms are still unknown.

IL-1β is an effective pro-inflammatory cytokine that causes the production of cytokines such as IL-6 and TNF-α (5). Importantly, IL-1β plays a critical role in modulating the immune response during both acute and chronic viral infection(6). The NLRP3 inflammasome is one of the best-characterized inflammasomes to urge IL-1β maturation. NLRP3 inflammasome activation requires NF-κB activation (priming signal) and NLRP3-ASC assembly (second signal). First, a priming signal is induced by pattern recognition receptors (PRRs). PPRs, such as RIG-I or MDA5, recognize viral nucleic acid and other molecular patterns and then induce NF-κB activation; NF-κB activation acts as a priming signal to initiate the transcription of pro-IL-1β and NLRP3 (7). The first signal is not enough to cause the production of IL-1β. The second signal is NLRP3 inflammasome assembly, consisting of NLRP3 and apoptosis-associated speck-like protein with a CARD domain (ASC) (8). The assembly of NLRP3-ASC inflammasome facilitates pro-Casp-1 cleavage into active subunits, which leads to pro-IL-1β splitting into a bioactive form for secretion (9) and three models of danger signal-induced NLRP3-ASC inflammasome assembly are widely supported: (i) the ion channel model (10); (ii) the lysosomal rupture model (11); and (iii) the reactive oxygen species (ROS) model (12).

The mechanisms of viral proteins for NLRP3 inflammasome activation were reported in previous studies. For example, the enterovirus 71 (EV 71) 3D protein associates with NLRP3 and enhances inflammasome complex assembly (13). The HCV core protein is a virion-specific factor in the activation of the NLRP3 inflammasome (14). Encephalomyocarditis virus (EMCV) viroporin 2B could induce the promptness of the NLRP3 inflammasome (15). Foot and mouth disease virus (FMDV) 2B protein triggers NLRP3 inflammasome activation (16). Zika virus NS5 facilitates NLRP3 activation by interacting with NLRP3 (17). Previous studies demonstrated that SVA infection could induce NF-κB-dependent inflammatory cytokines mRNA expression (18, 19). Although the NLRP3 inflammasome-mediated IL-1β production plays an essential role in viral infection, there are no mechanism studies on SVA that induce NF-κB and NLRP3 activation *in vitro* or *in vivo*. This study reveals a mechanism by which SVA activates pig IL-1β production by facilitating NLRP3 inflammasome assembly. Cellular- and animal-level studies showed that SVA facilitated IL-1β production in macrophages and pigs. SVA infection was shown to induce IL-1β production through the NLRP3 inflammasome. More significantly, we identified that SVA 3D polymerase interacts with IKKα and IKKβ to induce NF-κB activation, and 3D also directly interacts with the NATCH domain of NLRP3 using the N-terminus (amino acid [aa] 1 to 154) to facilitate NLRP3-ASC assembly, which induces IL-1β production. Moreover, 3D induced calcium influx and potassium efflux to activate the NLRP3 inflammasome at the second signaling step. Our study reveals that SVA infection regulates NLRP3 inflammasome complex assembly and provides a theoretical basis for the control of inflammation during SVA infection.

## RESULTS

### SVA infection induces IL-1β secretion and production.

**FIG 1:**
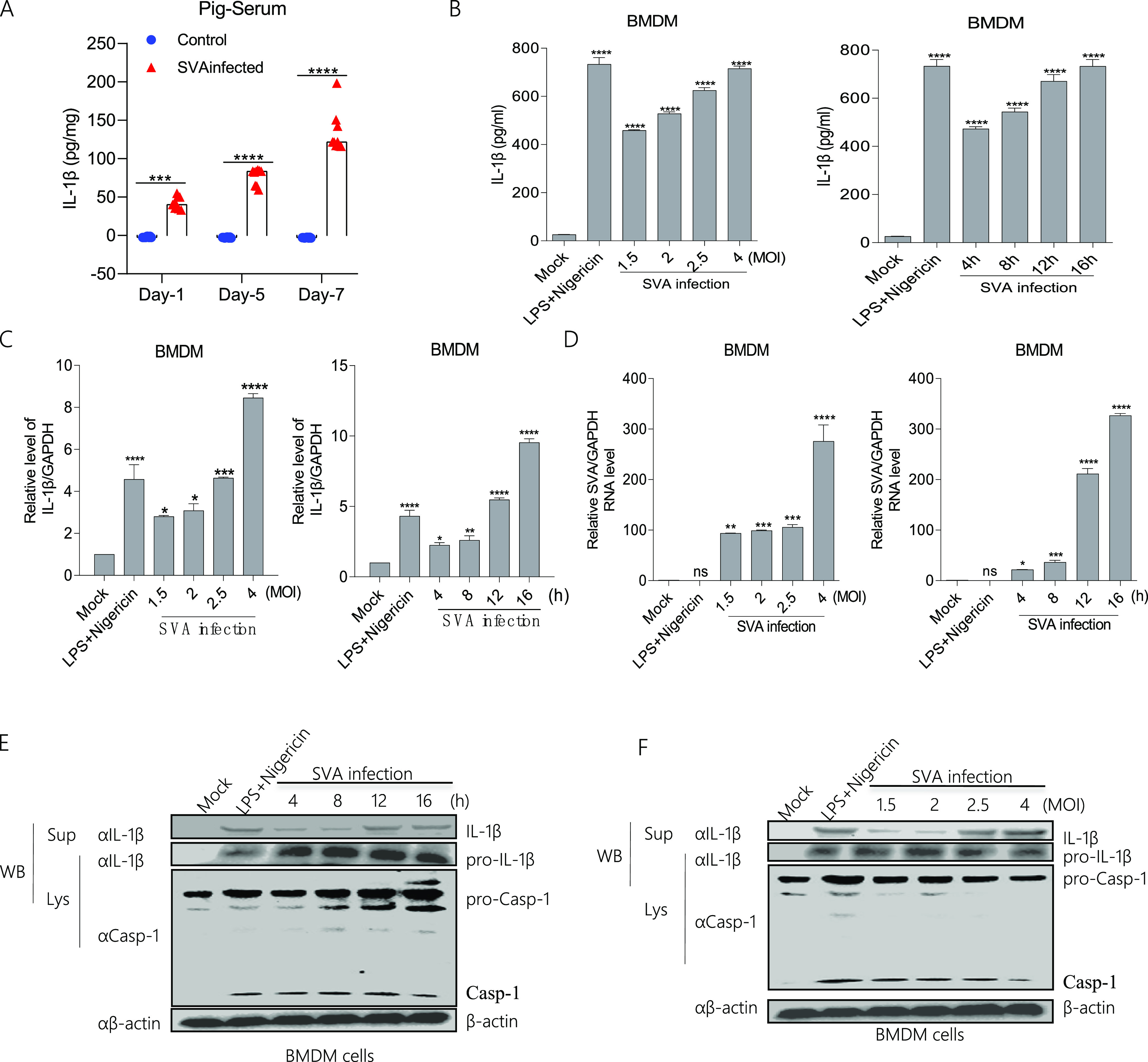
SVA infection induces IL-1β secretion and production. (A) The IL-1β levels of pig sera with (*n* = 9) or without (*n* = 9) SVA infection were detected by ELISA. Data shown are the mean±s.e.m.; ***, *P* < 0.001; **** < 0.0001 (one-way ANOVA with Tukey’s *post hoc* test). (B to D, E and F) BMDMs isolated from healthy pigs (3 months of old) were treated with 2 μM Nigericin for 2 h pre-primed with LPS (60 ng/mL) for 8 h or infected with SVA for 16 h at MOI = 1.5, 2, 2.5, 4, or at MOI = 4 for 4, 8, 12, 16 h. The IL-1β levels in the medium were detected by ELISA (B). The IL-1β mRNA levels and SVA mRNA levels at the indicated times and MOIs were determined by qPCR (C, D). IL-1β (17 kDa) expression in supernatants or pro-IL-1β (31 kDa), Caspase-1 (P20), and pro-casp-1 (45 kDa) expression in lysates were detected by Western blotting (E and F). For Western blot, the antibody dilution ratio was 1:1,000. The data shown are the mean±s.e.m; *, *P* < 0.05; **, *P* < 0.01; ***, *P* < 0.001; ****, *P* < 0.0001 versus mock (one-way ANOVA with Tukey's *post hoc* test). All experiments were repeated three times with similar results. Data were representative of the three independent experiments.

SVA infection induces clinical signs that suggest an inflammatory response. First, we determined the effect of SVA infection on IL-1β secretion and production in pigs. In the serum of SVA-infected pigs, IL-1β level increased during day 1 and day 7, and was significantly higher than in the controls (Fig. 1A). In pig bone marrow-derived macrophages (BMDMs) (Fig. 1B to D) as well as in human mononuclear phagocytes and the human monocytic cell line THP-1 (Fig. S1A and B), IL-1β secretion and mRNA transcription levels increased after lipopolysaccharide (LPS) along with Nigericin (NLRP3 activator) and SVA stimulation compared with those in the control conditions. By using Western blotting, we found that IL-1β and Casp-1 maturation form protein expression with LPS along with Nigericin and SVA stimulation increased at different hours postinfection (hpi) or multiplicity of infections (MOIs) in supernatants and cell lysates of bone marrow-derived macrophages (BMDMs) cells compared with that with control conditions (Fig. 1H and I). Taken together, the results showed that SVA could activate IL-1β production and secretion.

### SVA activates the NLRP3 inflammasome to induce IL-1β secretion.

**FIG 2:**
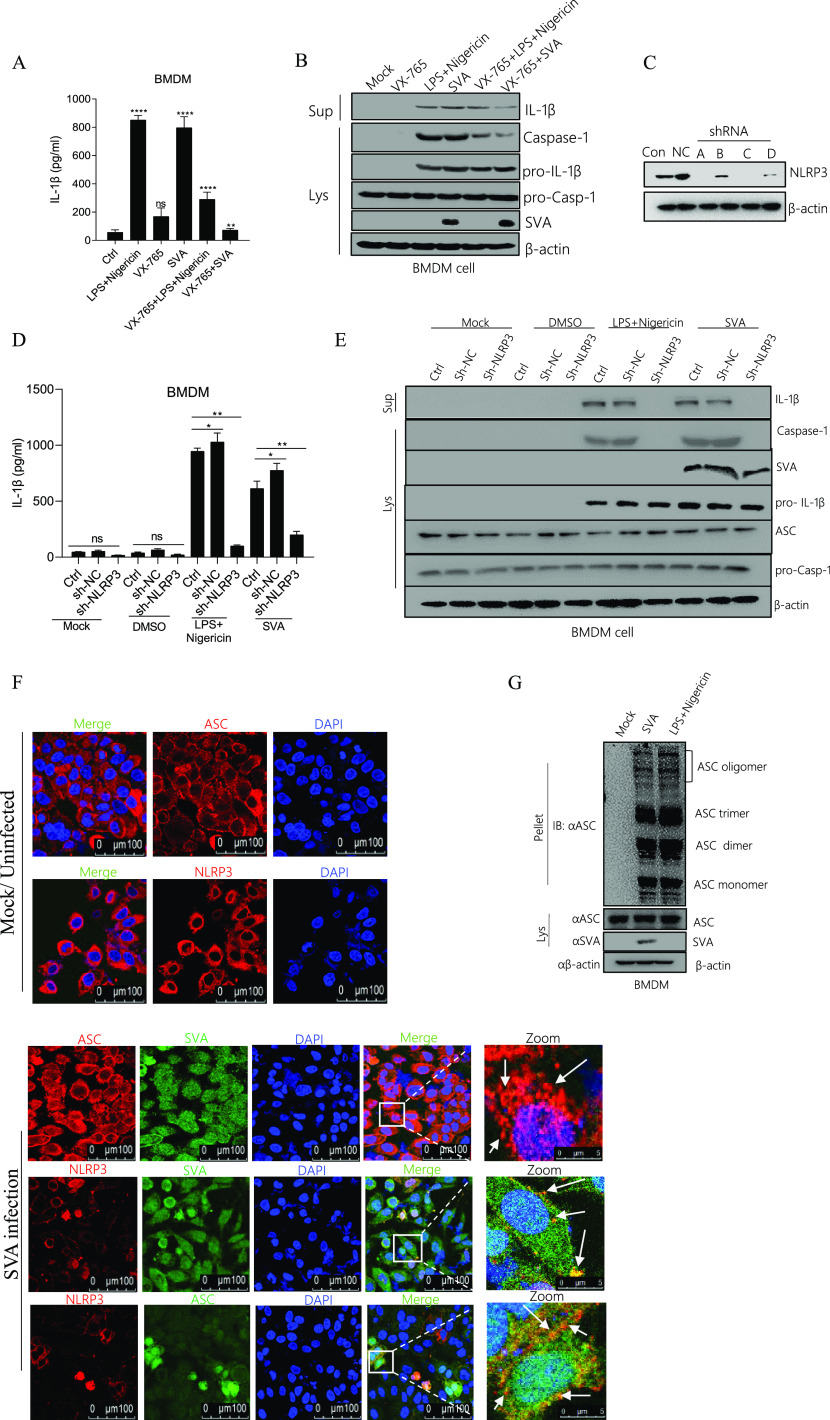
SVA activates the NLRP3 inflammasome to induce IL-1β secretion. (A, B) Porcine BMDM cells were treated with 2 μM Nigericin (NLRP3 stimulator) for 2 h pre-primed with LPS (60 ng/mL) for 8 h or 10uM VX-765 (casp-1 inhibitor) for 1 h or infected with SVA at MOI = 4 for 16 h or 10uM VX-765 together with 2 μM Nigericin and LPS (60 ng/mL) or 10uM VX-765 together with SVA infection. The IL-1β levels in the medium were determined by ELISA (A). IL-1β (17 kDa) expression in supernatants or pro-IL-1β (31 kDa), Caspase-1 (P20), and pro-casp-1 (45 kDa) expression in lysates were detected by Western blotting (B). (C) Porcine BMDM cells were infected with lentivirus-shRNAs targeting NLRP3 or sh-NC (negative control), and after 48 h, NLRP3 protein expression was detected by Western blotting. (D, E) Porcine BMDM cells were infected with lentivirus-shRNA targeting NLRP3 (shNLRP3-A) and treated with DMSO or 2 μM Nigericin for 2 h pre-primed with LPS (60 ng/mL) for 8 h or infected with SVA at MOI = 4 for 16 h. The IL-1β levels in the medium were determined by ELISA (D). IL-1β (17 kDa) expression in supernatants or pro-IL-1β (31 kDa), Caspase-1 (P20), ASC (22kDA), SVA, and pro-casp-1 (45 kDa) expression in lysates were detected by Western blotting (E). (F) Porcine BMDM cells were infected with SVA at MOI = 4 for 16 h. NLRP3, ASC, and SVA subcellular localizations were assayed by confocal microscopy. The scale bar was 100 μm, and the zoom section was 5 μm. (G) Porcine BMDM cells were infected with SVA at MOI = 4 for 16 h or treated with 2 μM Nigericin for 2 h pre-primed with LPS (60 ng/ml0 for 8 h). ASC oligomerization with ASC primary antibody was detected by Western blotting. For Western blot, the antibody dilution ratio was 1:1,000. The data shown are the mean±s.e.m. *, *P* < 0.05; **, *P* < 0.01; ***, *P* < 0.001; ****, *P* < 0.0001 versus mock (one-way ANOVA with Tukey's *post hoc* test). All experiments were repeated three times with similar results. Data were representative of the three independent experiments.

Casp-1 controls the production of the maturation of IL-1β (20, 21). To address whether Casp-1 is involved in SVA-induced IL-1β production, we assessed IL-1β secretion and IL-1β maturation form expression in BMDM cells after SVA infection. LPS along with Nigericin or SVA infection could induce IL-1β secretion and IL-1β maturation, but VX-765 (Casp-1 inhibitor) inhibited SVA-induced IL-1β production (Fig. 2A and B). The data demonstrated that Casp-1 is involved in the activation of IL-1β during SVA infection. Next, to understand the role of SVA in NLRP3-mediated IL-1β production after SVA infection, we first constructed four types of short-hairpin RNA (shRNA) which target NLRP3, as shown in Fig. 2C, number A and C shRNA effectively interfere with the expression of NLRP3 compared with Mock and NLRP3 shRNA negative control (NC) (lane NC). Then we observed IL-1β secretion and maturation, but short hairpin RNA of NLRP3 (sh-NLRP3-A) attenuated these activations in BMDM (Fig. 2D and E). Thus, the knockdown of inflammatory components of NLRP3 decayed IL-1β activation during SVA infection. The formation of ASC specks is a direct indicator of the dynamic development of the NLRP3 inflammasome (22). We showed that NLRP3 and ASC were dispersed in the cytoplasm in the non-infected state but formed tiny NLRP3-ASC specks when infected by SVA (Fig. 2F), indicating that SVA activates the inflammasome complex to promote NLRP3-ASC speck formation. ASC oligomerization is another substantial measure of inflammasome activation. Furthermore, we observed that LPS along with Nigericin and SVA promoted ASC oligomerization in BMDM cells (Fig. 2G). Taken together, the results suggested that SVA affects NLRP3 inflammasome activation to induce IL-1β secretion.

### SVA RNA and proteins are associated with NLRP3 activation.

**FIG 3:**
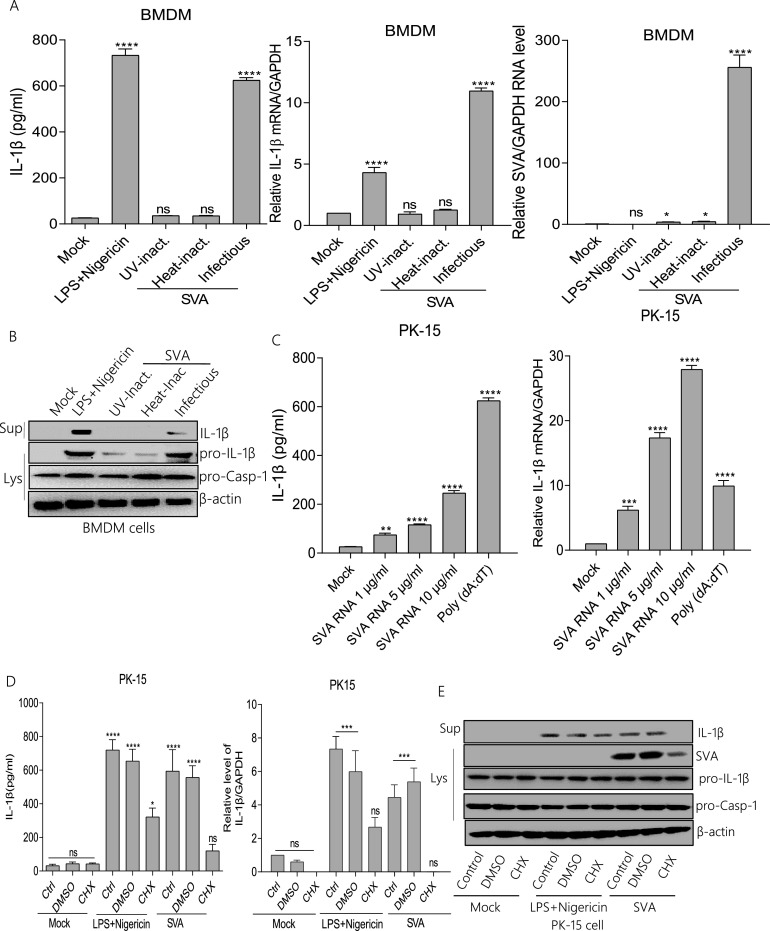
SVA RNA and proteins are associated with NLRP3 activation. (A, B) Porcine BMDMs cells were treated with 2 μM Nigericin for 2 h pre-primed with LPS (60 ng/mL) for 8 h or inoculated with UV-inactivated SVA, heat-inactivated SVA, or infected with SVA (MOI = 4) for16 h. IL-1β levels from medium or IL-1β mRNA and SVA mRNA levels were detected by ELISA and qPCR (A). IL-1β (17 kDa) expression in supernatants or pro-IL-1β (31 kDa) and pro-casp-1 (45 kDa) expression in lysates were detected by Western blotting (B). (C) PK-15 cells were treated with Lipo, stimulated with Lipo plus poly (dA:dT), or treated with Lipo and transfected with 1, 5, or 10 μg/mL genomic RNA of SVA for 24 h. IL-1β levels from medium or IL-1β mRNA levels were detected by ELISA and qPCR. (D, E) PK-15 cells were treated with 2 μM Nigericin for 2 h pre-primed with LPS (60 ng/mL) for 8 h, 100 μM CHX (translation inhibitor) for 1 h, or infected with SVA (MOI = 4) for 16 h. IL-1β levels from medium or IL-1β mRNA levels were detected by ELISA and qPCR (D). IL-1β (17 kDa) expression in supernatants or pro-IL-1β (31 kDa), SVA, and pro-casp-1 (45 kDa) expression in lysates were detected by Western blotting (E). After transfection, samples were harvested for 24 h. For Western blot, the antibody dilution ratio was 1:1,000. The data shown are the mean±s.e.m. *, *P* < 0.05; **, *P* < 0.01; ***, *P* < 0.001; ****, *P* < 0.0001 versus mock (one-way ANOVA with Tukey's *post hoc* test). All experiments were repeated three times with similar results. Data were representative of the three independent experiments.

The impacts of SVA replication and translation on IL-1β production were investigated. As shown in Fig. 3A and B, in BMDM cells, IL-1β secretion and mRNA expression were induced with LPS along with Nigericin and SVA stimulation but not UV-inactivated or heat-inactivated SVA. The cleavage of IL-1β was also observed in the supernatants BMDM cells (Fig. 3B). Because SVA failed to replicate or infect after UV or heat treatment, these data suggested that SVA replication or infection is essential for IL-1β activation. To determine whether SVA RNA is involved in IL-1β activation, we transfected the SVA RNA into the PK-15 cells. We observed that SVA genomic RNA is involved in IL-1β secretion and mRNA expression. Poly(dA:dT) (an inflammasome activator) as a positive control also induced IL-1β production (Fig. 3C). These results suggested that SVA RNA is involved in IL-1β activation. Another study showed that cycloheximide (CHX)-pretreated cells significantly inhibited EMCV-induced IL-1β production (15). Therefore, we wondered whether protein synthesis affects NLRP3 inflammasome-mediated IL-1β activation. As shown in PK-15, LPS along with Nigericin and SVA induced IL-1β secretion and transcription in the absence of CHX (Fig. 3D). Simultaneously, IL-1β cleavage disappeared in the presence of CHX (Fig. 3E). These data indicated that the transcription of SVA proteins is vital for activating NLRP3-mediated IL-1β production. Taken together, these results show that NLRP3 inflammasome-mediated IL-1β activation requires SVA infection, SVA genomic RNA, and protein synthesis.

### SVA polymerase 3D facilitates NLRP3-mediated IL-1β production.

**FIG 4:**
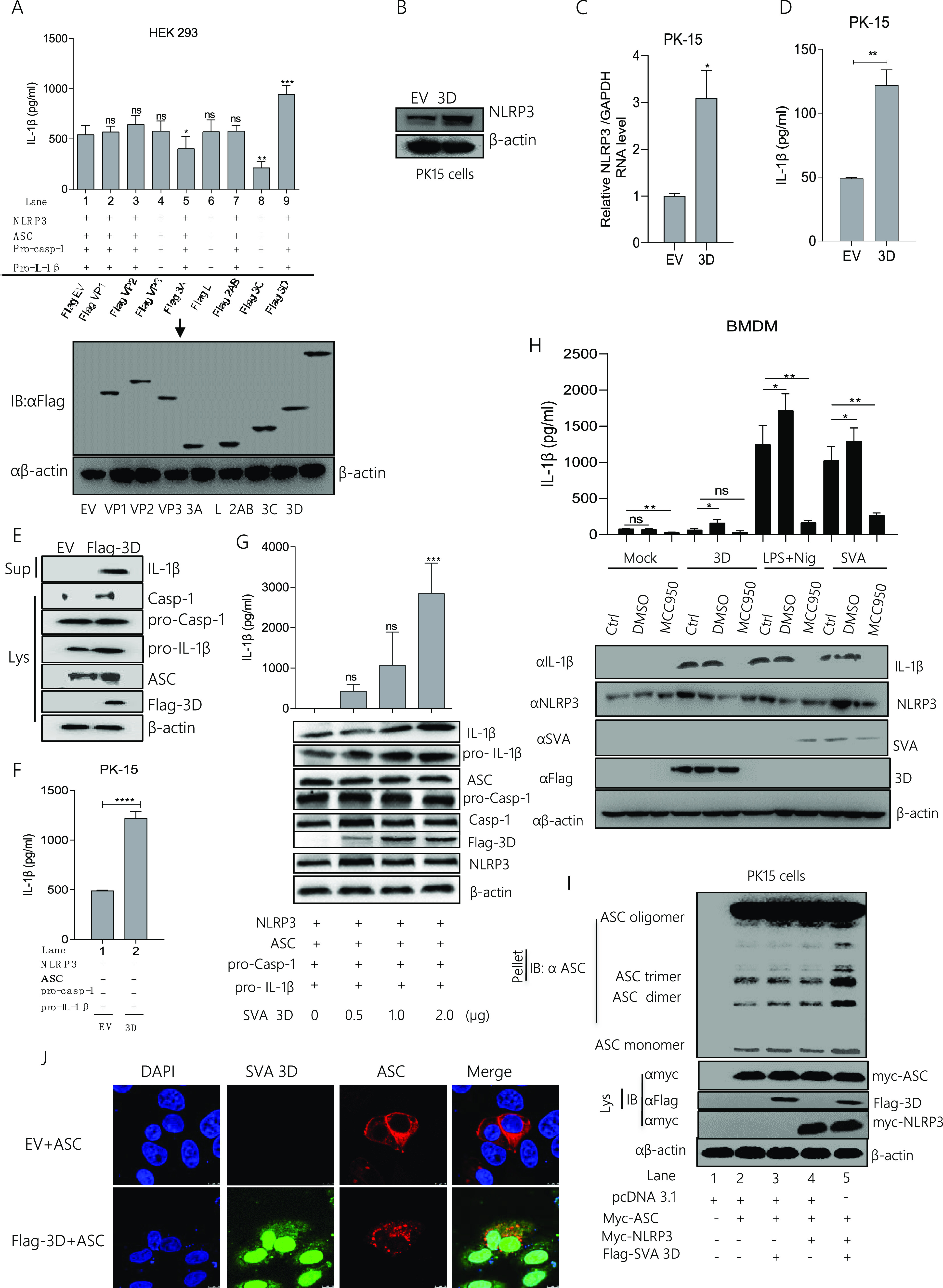
SVA polymerase 3D facilitates NLRP3-mediated IL-1β production. (A) HEK293 cells were co-transfected with 2 μg of plasmids encoding NLRP3, ASC, pro-casp-1, and pro-IL-1β. The cells were then transfected with 2 μg of pcDNA 3.1 as an empty vector, Flag VP1, Flag VP2, Flag VP3, Flag 3A, Flag L, Flag 2AB, Flag 3C, and Flag 3D separately with the above-indicated plasmids. IL-1β levels from the medium were detected by ELISA, and all the SVA plasmids (Flag VP1, Flag VP2, Flag VP3, Flag 3A, Flag L, Flag 2AB, Flag 3C, and Flag 3D) expressions were detected by Western blotting (A). (B to E) PK-15 cells were transfected with 2 μg Flag-3D or 2 μg empty vector pcDNA 3.1. Cell lysates were subjected to SDS-PAGE, NLRP3 (110 kDa) was detected by Western blotting (B), and NLRP3 mRNA levels were detected qPCR (C), IL-1β levels from medium were detected by ELISA (D). IL-1β (17 kDa) expression in supernatants or Caspase-1 (P20), pro-IL-1β (31 kDa), ASC (22kDA), Flag 3D (51 kDa), and pro-casp-1 (45 kDa) expression in lysates were detected by Western blotting (E). (F) PK-15 cells were co-transfected with 2 μg plasmids encoding NLRP3, ASC, pro-casp-1, and pro-IL-1β and then transfected with 2 μg Flag 3D or 2 μg empty vector pcDNA 3.1. IL-1β levels from the medium were detected by ELISA. (G) PK-15 cells were co-transfected with NLRP3, ASC, pro-casp-1, and pro-IL-1β and transfected with plasmids expressing the Flag 3D of SVA in a dose-dependent manner (0, 0.5, 1.0, 2.0 μg). IL-1β levels were detected by ELISA and IL-1β (17 kDa) expression in supernatants or pro-IL-1β (31 kDa), ASC (22 kDa), Flag 3D (51 kDa), mature Caspase-1 (20 kDa), NLRP3 (110 kDa), and pro-casp-1 (45 kDa) expression in lysates were detected by Western blotting. (H) The BMDM cells were transfected with 2 μg of 3D, postinfected with SVA or post-stimulated with LPS (60 ng/mL) and Nigericin (2 μM), and the cells were untreated or treated with DMSO or 5 μM MCC950 (NLRP3 activation inhibitor) for 16 h. Cell lysates were subjected to Western blotting, IL-1β (17 kDa) expression in supernatants or NLRP3 (110 kDa), SVA, and Flag 3D (51 kDa) expression in lysates were detected by Western blotting and also IL-1β levels from medium were detected by ELISA. (I) PK-15 cells were co-expressed with 2 μg of pcDNA 3.1, myc-ASC, myc-NLRP3, or Flag 3D. Cell lysates and pellets were subjected to ASC oligomerization detection. (J) PK-15 cells were transfected with EV+ ASC, Flag-3D+ ASC. Subcellular localization was observed by confocal microscopy, and the scale bar is 10 μm. After transfection, samples were harvested for 24 h. For Western blot, the antibody dilution ratio was 1:1,000. The number of replicates is three. Data shown are mean±s.e.m.; *, *P* < 0.05; **, *P* < 0.01; ***, *P* < 0.001; ****, *P* < 0.0001 vs EV; ns, no significance (one-way ANOVA with Tukey’s *post hoc* test).

To determine whether the SVA protein is required to activate NLRP3 inflammasome-mediated IL-1β activation, NLRP3, ASC, pro-Casp-1, and pro-IL-1β were expressed together to establish an IL-1β activation cell system and then co-expressed with each of the seven SVA proteins. Our results showed that the 3D protein, but not the others, promoted IL-1β secretion compared with control (EV transfection). It suggested that 3D is involved in promoting NLRP3 inflammasome-mediated IL-1β secretion (Fig. 4A). We also detected NLRP3 protein and mRNA expression after 3D transfection (Fig. 4B and C). The data in Fig. 4B and C showed that 3D increased NLRP3 expression. Moreover, to determine whether 3D can induce IL-1β secretion, the data as shown in Fig. 4D and 4E, 3D can induce the IL-1β protein secretion and expression. Meanwhile, as shown in Fig. 4F, we found 3D increases the IL-1β secretion in an IL-1β production system. At the same time, IL-1β secretion was increased in a dose-dependent manner after 3D expression, indicating an essential role of 3D in the activation of NLRP3-mediated IL-1β production (Fig. 4G). Next, we observed robust IL-1β secretion and maturation induced by 3D expression, but NLRP3 activation inhibitor (MCC950) attenuated these activations in BMDM (Fig. 4H). Thus, the knockdown of inflammatory components of NLRP3 decayed IL-1β activation during SVA infection. In addition, we assessed the effect of 3D on ASC polymer formation. ASC oligomerization can be formed after ASC transfection without NLRP3 and viral protein stimulus (17). Our study showed that, in the presence of ASC, the ASC oligomerization was formed, but ASC oligomerization was enhanced in the presence of both 3D and NLRP3 (Fig. 4I). Furthermore, we showed that ASC formed tiny specks when 3D expression (Fig. 4J), indicating that 3D activates the inflammasome to promote ASC speck formation. In combination with NLRP3, ASC undergoes conformational modification to form an active inflammasome complex (23). These results indicated that 3D increased NLRP3 inflammasome mediated IL-1β production.

### The SVA 3D N-terminus (aa 1 to 154) binds with the NACHT domain of NLRP3 to facilitate NLRP3 inflammasome assembly and activation.

**FIG 5:**
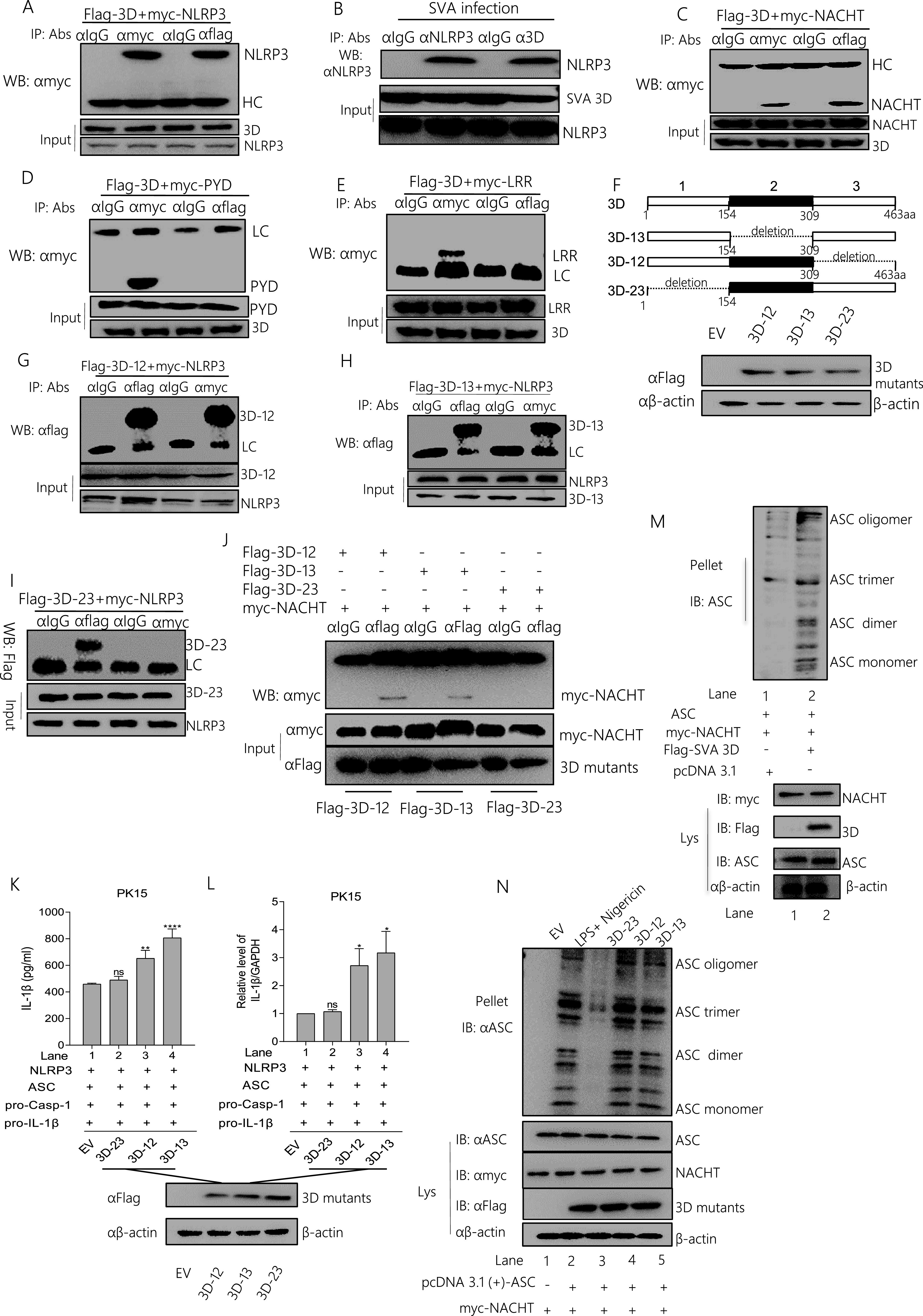
The SVA 3D N-terminus (1 to 154 amino acids) binds with the NACHT domain of NLRP3 to facilitate NLRP3 inflammasome assembly and activation. (A, C to E) PK-15 cells were transfected with 5 μg Flag-3D along with the 5 μg myc-NLRP3, myc-NACHT, myc-PYD, or myc-LRR- the domain of NLRP3. Cell lysates were subjected to IP using IgG, anti-myc, or anti-Flag primary antibody and detected with Western blotting using the indicated antibody (WB). The protein samples as input were subjected to Western blotting. (B) PK-15 cells were infected with SVA. Cell lysates were subjected to IP using IgG, anti-NLRP3, or anti-SVA 3D primary antibody and detected with Western blotting using the NLRP3 antibody (WB), or the protein samples as input were subjected to Western blotting. (F)The 3D gene was divided into three segments of average length, identified as Flag 3D-12 (Δ309-463 aa), Flag-3D-13 (Δ154 to 309 aa), and Flag-3D-23 (Δ1 to 154 aa), and each segment was deleted separately. PK-15 cells were transfected with 2 μg Flag-3D-12 (34 kDa), Flag-3D-13 (34 kDa), Flag-3D-23 (34 kDa), cell lysates were subjected to SDS-PAGE and detected the 3D segments. (G to J) 5 μg of the SVA 3D deletion mutants (Flag-3D-13, Flag-3D-12, Flag-3D-23) co-expressed with 5 μg myc-NLRP3 or myc-NACHT. After 24 h, protein samples were subjected to IP with the indicated antibody and detected by Western blotting. (K to L) 2 μg of Flag-3D deletion mutants (Flag 3D-12, Flag-3D-13, Flag-3D-23) were co-transfected with 2 μg NLRP3, ASC, pro-Casp-1, and pro-IL-β in PK-15 cells. The IL-1β levels from medium and IL-1β mRNA levels were determined by ELISA and qPCR. (M to N) PK-15 cells were co-expressed with 2 μg pcDNA 3.1, ASC, myc-NACHT, along with Flag 3D (M) or Flag-3D mutants (N). Cell lysates and pellets were subjected to ASC oligomerization detection. After transfection, samples were harvested for 24 h. For Western blot, the antibody dilution ratio was 1:1,000. Data shown are mean±s.e.m.; *, *P* < 0.05; **, *P* < 0.01; ***, *P* < 0.001; ****, *P* < 0.0001 vs EV; ns, no significance (one-way ANOVA with Tukey’s *post hoc* test). All experiments were repeated three times with similar results. Data were representative of the three independent experiments.

Next, the mechanism by which 3D stimulates NLRP3 inflammasome-mediated IL-1β production was further studied. We found that 3D promoted NLRP3 expression (Fig. 4B and C). Therefore, we speculate that 3D may affect the function of NLRP3. As seen in Fig. 5A and Fig. S2A, we observed that 3D interacts with NLRP3, indicating that 3D promotes IL-1β production by interacting with the NLRP3. At the same time, we infected the PK-15 cells with SVA and used IgG, NLRP3, or SVA 3D as the IP primary antibody; we found 3D interacts with endogenous NLRP3 (Fig. 5B and Fig. S2B). NLRP3 consists of PYRIN, NACHT, and seven LRR domains (24). As shown in Fig. 5C to E and S2C-S2E, 3D directly interacted with the NACHT domain of NLRP3 but not LRR or PYRIN. Deletion mutants of 3D were constructed, which were divided into an average of three sections (Fig. 5F). Using co-IP detection, as shown in Fig. 5G to I and S2F-S2H, the N-terminus (aa 1 to 154) of 3D is associated with NLRP3 but not with the middle part or the C-terminus deletion mutant. Moreover, the N-terminus (aa 1 to 154) of 3D interacted with the NACHT domain of NLRP3 (Fig. 5J and Fig. S2I). These data suggested that the N-terminus (aa 1 to 154) of 3D binds NLRP3 by interacting with the NACHT domains. To assess the effect of the N-terminus of 3D on IL-1β production, we determined that the N-terminus of 3D (aa 1 to 154), together with NLRP3, ASC, pro-caspase-1, and pro-IL-1β, increased IL-1β secretion compared with the control (Fig. 5K). Similarly, the IL-1β mRNA level was increased after transfection of the N-terminus of 3D (aa 1 to 154) (Fig. 5L). The effect of 3D and its mutants on ASC polymer formation was assessed, the results showed that ASC oligomerization was enhanced in the presence of both 3D, 3D-1-2, or 3D-1-3, and NACHT (Fig. 5M and N). These data suggested that the 3D N terminal plays an essential role in NLRP3 inflammasome activation.

**FIG 6:**
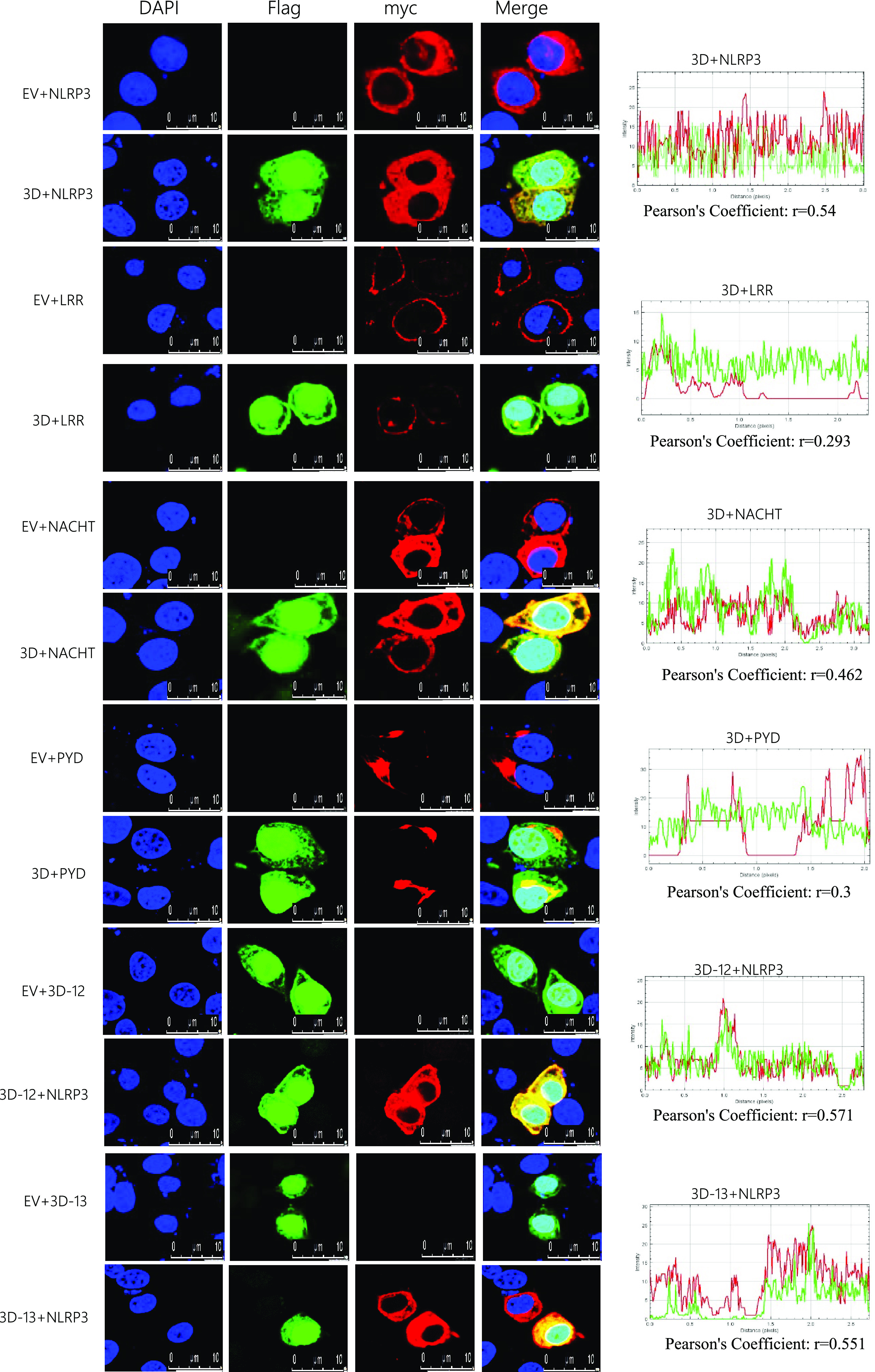
Co-localization of SVA 3D mutants with NLRP3 domains. PK-15 cells were transfected with EV together with Myc-NLRP3, Flag-3D together with myc-NLRP3, EV together with Myc-LRR, Flag-3D together with myc-LRR, EV together with Myc-NACHT, Flag-3D together with Myc-NACHT, EV together with Myc-PYD, Flag-3D together with Myc-PYD, EV together with Flag-3D-12, Flag-3D-12 together with Myc-NLRP3, EV together with Flag-3D-13, and Flag-3D-13 together with Myc-NLRP3. Subcellular localization was observed by confocal microscopy. The Pearson’s correlation coefficient was analyzed using the Image-J (Java 1.8.0_172) software. The scale bar was 10 μm.

Because SVA polymerase 3D targets NLRP3, the cell biological effects of 3D and NLRP3 co-expression were investigated. Single expressed 3D protein clustered in both cytoplasm and nucleus or NLRP3 clustered in the cytoplasm. Then, the sublocation of the 3D and NLRP3 fragments was assessed with confocal microscopy. As shown in Fig. 6, 3D or 3D mutants were colocalized with NLRP3, NATCH, PYD, and LRR in the cytoplasm (Fig. 6). Although 3D is also colocalized with PYD and LRR domain, Pearson’s correlation coefficient (PCC) was used to quantify the degree of co-localization between interacting partners. We found that the PCC value of 3D with PYD or LRR is much lower than the 3D with NLRP3 or NATCH.

Taken together, these results showed that the N-terminus (aa 1 to 154) of 3D promotes the assembly of the NLRP3 complex inflammasome by directly interacting with NLRP3 NACHT domains.

### SVA 3D induces NLRP3-mediated IL-1β production that depends on the NF-κB pathway and ion channels.

**FIG 7:**
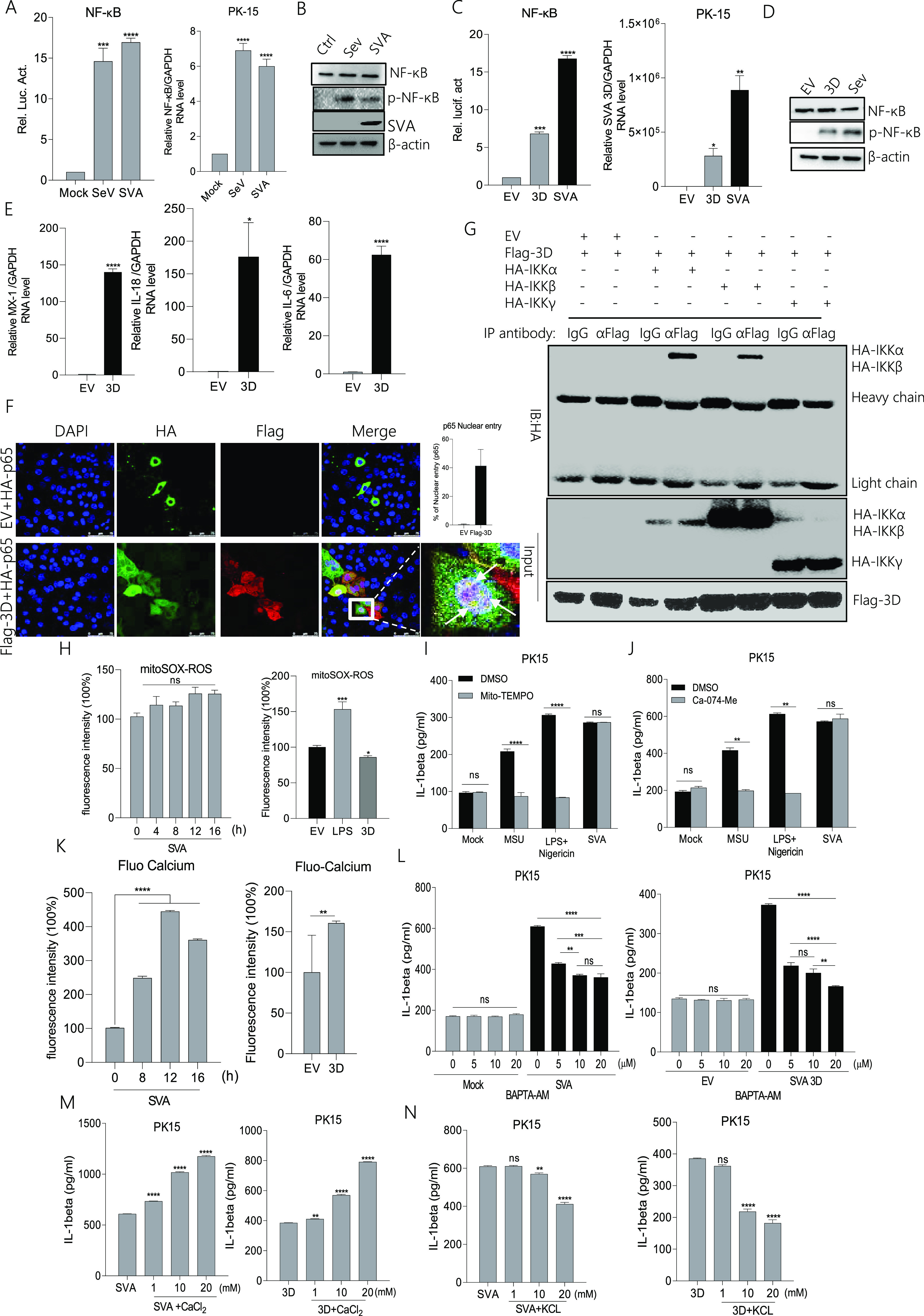
SVA 3D-induced NLRP3 activation depends on the NF-κB pathway and ion channels (A, C) PK-15 cells were transfected with 2 μg of NF-κB luciferase reporter plasmid (A) or together with 2 μg of 3D (C); after 24 h, cells were infected with SeV or SVA, and controls were kept uninfected. The cell lysates were subjected to a dual-luciferase assay. (A to D) PK-15 cells were infected with SeV or SVA or transfected with 2 μg of EV or 3D. NF-κB mRNA levels and SVA 3D mRNA levels were detected by qPCR (A, C), and NF-κB (P65) and p- NF-κB (P-P65) proteins were detected by Western blotting (B, D). (E) PK-15 cells were transfected with 2 μg of Flag-3D or empty vector pcDNA 3.1, MX-1, IL-18, and IL-6 levels were determined by qPCR. (F) PK-15 cells were transfected with EV+ HA-p65, Flag-3D+ HA-p65. Subcellular localization was observed by confocal microscopy, and the scale bar was 75 μm. (G) PK-15 cells were transfected with 5 μg Flag-3D along with the 5 μg HA-IKKα, HA-IKKβ, and HA-IKKγ. Cell lysates were subjected to IP using IgG, anti-HA, or anti-Flag primary antibody and detected with Western blotting using the indicated antibody (WB). The protein samples as input were subjected to Western blotting. (H) PK-15 cells were infected with SVA or transfected with 2 μg 3D were treated with 5 μM mito-SOX for 10 min at 37°C. The cell lysates were collected at the indicated time points, and the fluorescence intensity was analyzed by flow cytometry. A total of 40,000 cells were counted. (I to J) PK-15 cells were treated or untreated with MSU (2.5 mM), LPS (60 ng/mL)+Nigericin (2 μM), and SVA in the presence of DMSO, Mito-TEMPO (500 μM), or Ca-074-Me (10 μM). IL-β levels were determined by ELISA. (K) PK-15 cells were infected with SVA or transfected with 2 μg of 3D and treated with 1 μM Fluo-3 AM for 1 h at 37°C. The cell lysates were collected at the indicated time points, and the fluorescence intensity was analyzed by flow cytometry. A total of 40,000 cells were counted. (L to N) PK-15 cells infected with SVA or transfected with 3D were treated with BAPTA-AM, CaCl_2_, or KCl at the indicated doses. IL-β levels were determined by ELISA. After transfection, samples were harvested for 24 h. For Western blot, the antibody dilution ratio was 1:1,000. Data are presented as the mean ± s.e.m. of triplicate measurements in three independent experiments. ns: not significant, *, *P* < 0.05; **, *P* < 0.01; ***, *P* < 0.001; ****, *P* < 0.0001 vs EV, mock or SVA (one-way ANOVA with Tukey’s *post hoc* test). The results were analyzed by flow cytometric analysis with FlowJo software V10.

NLRP3 activation involves NF-κB-dependent transcription of NLRP3 and pro-IL-1β as the initial priming phase (“signal 1”) via stimulation of pathogens (25, 26). Thus, we tested the effect of SVA or 3D on NF-κB activation. As shown in Fig. 7A, using luciferase reporter gene detection, we found that SVA significantly increased NF-κB promoter activity compared with the Mock (Sendai virus, SeV as a positive control). The NF-κB mRNA levels also increased after SVA infection compared with Mock. Simultaneously, NF-κB was phosphorylated after SVA infection (Fig. 7B). These data suggested that SVA induced “signal 1” activation. Similar to the SVA infection, 3D over-expression significantly increased NF-κB promoter activity compared with the control (Fig. 7C). Meanwhile, 3D also increased NF-κB phosphorylation (Fig. 7D). NF-κB activation could induce multiple cytokines mRNA transcription; we determined the effect of 3D on downstream cytokines expression of NF-κB. As shown in Fig. 7E, 3D increases the cytokines mRNA expression, such as Mx1, IL-18, and IL-6. The p65 also entered into the nucleus after 3D over-expression, and compared with the control, the proportion of cells in which p65 protein translocated into the nucleus was significantly increased after 3D expression(Fig. 7F). It indicated that 3D induced NF-κB activation. Moreover, coimmunoprecipitation assay (Co-IP) experiment result showed in Fig. 7G and Fig. S3A that 3D interacted with IKKα and IKKβ, it suggested that 3D might induce NF-κB activation through interaction with IKKα and IKKβ. These results suggested that SVA and 3D could induce “signal 1” of NLRP3 inflammasome activation. The second phase of NLRP3 activation depends on ROS, lysosomes, or ion channels (27–29). Next, we assessed the effect of 3D on “signal 2” of NLRP3 activation. We found that the ROS levels were not different from those of the control during SVA infection. Interestingly, 3D decreased cell basic ROS production; LPS was used as a positive stimulator (Fig. 7H). Monosodium urate (MSU) and Nigericin are activators of NLRP3 (30, 31). Mito-TEMPO is an antioxidant that inhibits ROS as an inhibitor of NLRP3 (32, 33). As shown in Fig. 7I, there was no effect on IL-1β secretion in SVA-infected cells treated with Mito-TEMPO, but IL-1β secretion was significantly inhibited in response to Nigericin or MSU. These results suggested that SVA-induced NLRP3 activation does not depend on the ROS model.

The lysosomal model focuses on the cathepsin B protease, which activates inflammatory NLRP3 (34). As shown in Fig. 7J, treatment with the specific cathepsin B inhibitor Ca-074-Me had no effect on SVA-induced IL-1β secretion but decreased the MSU or Nigericin effect. These results suggested that NLRP3 activation induced by SVA was not dependent on the lysosome model.

Because cytosolic K^+^ efflux and Ca^2+^ influx are important for triggering NLRP3 activation (9, 35), the intracellular K^+^ and Ca^2+^ levels were measured after SVA or 3D treatment. SVA infection or 3D transfection was followed by staining with the calcium-dependent fluorescent dye Fluo-3 AM. As shown in Fig. 7K, the concentration levels of intracellular Ca^2+^ increased significantly after SVA infection or 3D expression. As seen in Fig. 7L, cells treated with the cell-permeant Ca^2+^ chelator BAPTA-AM in a dose-dependent manner exhibited a significant decrease in IL-1β secretion during SVA infection or 3D expression. A similar result was observed in Fig. 7M. SVA- or 3D-induced IL-1β secretion was increased after incubation with gradient concentrations of CaCl_2_. Simultaneously, as shown in Fig. 7N, IL-1β secretion was inhibited in SVA-infected or 3D-expressing cells treated with KCl at gradient concentrations. These results suggested that SVA- or 3D-induced NLRP3 activation depended on Ca^2+^ influx and K^+^ efflux.

### SVA 3D induced an inflammatory response in animals.

**FIG 8:**
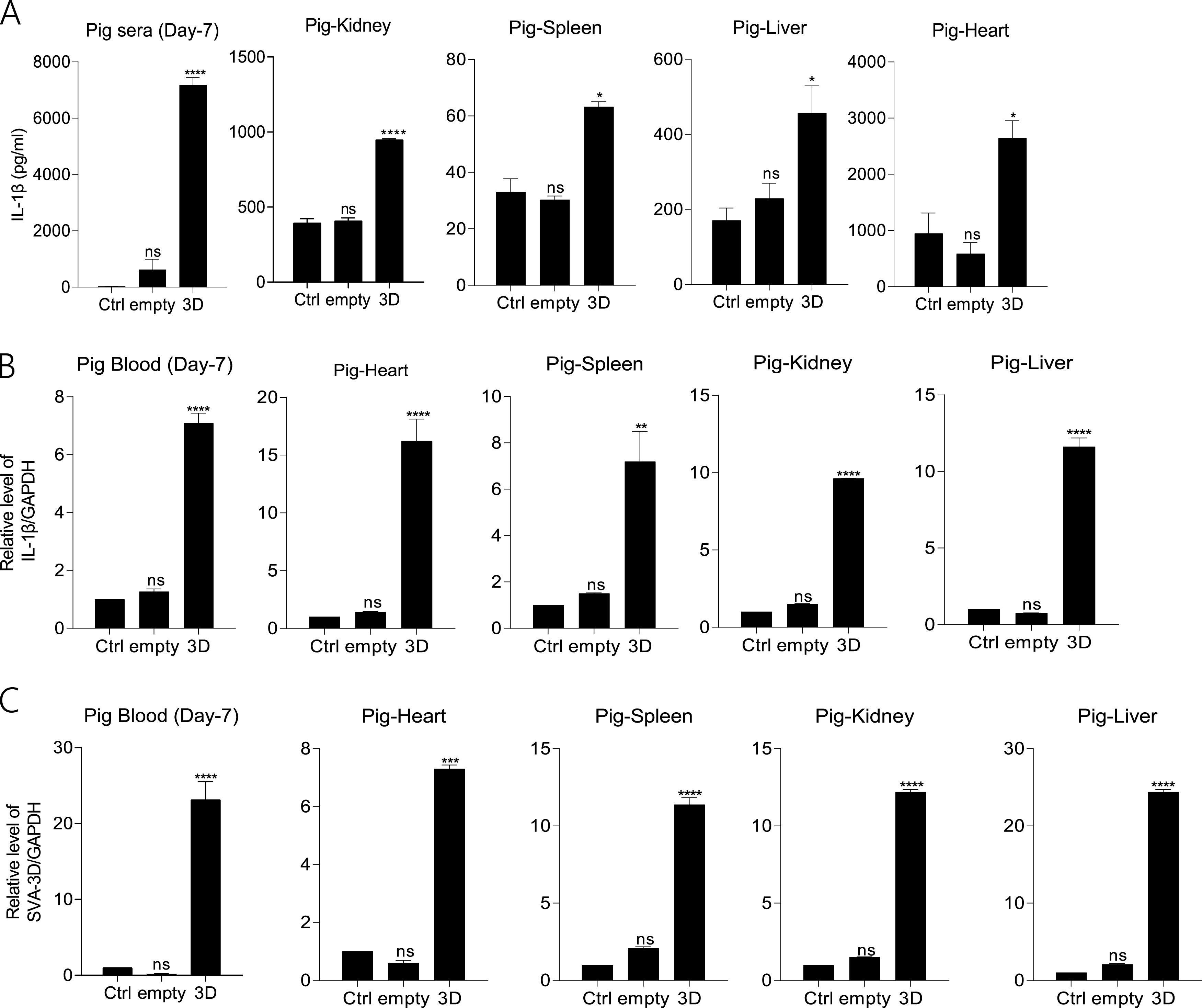
SVA 3D induced an inflammatory response in animals. (A to C) Pigs (3 months old) were treated with DMEM or infected with empty lentivirus or 3D expression lentivirus (1 × 10^8^ TU/mL) (each of them *n* = 3) by intramuscular injection for 7 days. The levels of IL-1β in pig organs (sera, liver, kidney, spleen, and heart) were determined by ELISA and qPCR (A, B), and SVA 3D mRNA levels were detected by qPCR (C). The data shown are the mean±s.e.m; *, *P* < 0.05; **, *P* < 0.01, ***, *P* < 0.001; ****, *P* < 0.0001 vs Ctrl (one-way ANOVA with Tukey’s *post hoc* test). All experiments were repeated three times with similar results. Data were representative of the three independent experiments.

The biological effects of 3D on inflammatory responses were investigated in natural pig hosts. The pigs were injected with Dulbecco’s modified Eagle’s medium (DMEM), empty lentivirus, and recombinant 3D lentivirus. IL-1β secretion was detected in serum and organs. The results shown in Fig. 8A indicate that 3D expression induced IL-1β secretion in serum and organs. After 3D expression, the IL-1β mRNA levels were consistently higher than those in control in spleen, heart, liver and kidney tissues (Fig. 8B). We found 3D mRNA transcription in recombinant 3D lentivirus-infected pigs (Fig. 8C).

Mice were also injected with DMEM, empty lentivirus, and recombinant 3D lentivirus. From Fig. S4A, we found the organs of the heart, kidney, spleen, and liver were congested and swelled. ELISA detection in the serum and organs showed that IL-1β secretion was increased after 3D protein expression compared with the control group. Similar to the results of IL-1β secretion, IL-1β mRNA transcription and protein expression was also increased compared with the control group (Fig. S4B). In Fig. S4C, hematoxylin and eosin (H&E) staining showed that the interstitial space of myocardial cells changed massively (black arrow), a small amount of inflammatory cell diffusion (yellow arrow) was observed in the stroma; the scattered inflammatory cell infiltration (yellow arrow) was seen in the local endocardium; the atrial congestion and dilation were observed in the tissue of lenti-3D injected mice. After 3D protein induction, we also found from the kidney that a large number of renal tubules were loose in structure, and the cytoplasm of epithelial cells was loose and light stained (black arrow); many renal tubular epithelial cells were necrotic (blue arrow), and the nucleus was pyknotic; extensive interstitial congestion occurred (green arrow). Slightly increased white blood cells in hepatic sinusoids (yellow arrow) were observed in 3D expressed mice liver at 7 days. Meanwhile, moderate bleeding of the red pulp (blue arrow) was also observed in the spleen of 3D protein-expressed mice.

These results proved that the 3D protein induces the inflammatory response in pigs. In summary, we revealed that SVA infection and 3D expression induce an inflammatory response in animals.

## DISCUSSION

SVA causes fever, diarrhea, vesicles and erosions, and even death. Therefore, SVA can induce significant inflammatory clinical symptoms. The innate immune system is the first line of defense against invading pathogens (36). IL-1β production and secretion are essential components of inflammation of the innate immune response associated with viral infection (37, 38). IL-1β production is tightly regulated by the NLRP3 inflammasome complex, which consists of NLRP3 with ASC to recognize danger signals to promote pro-Casp-1 cleavage and regulate IL-1β maturation, ultimately leading to inflammation (9, 21, 39).

The NLRP3-mediated inflammasome is activated by viruses, such as the Zika virus (17), EMCV (15, 40), enterovirus 71 (EV71) (13), and foot and mouth disease virus (FMDV) (16). In this study, we proved that SVA infection induces IL-1β secretion in macrophages and pigs. Notably, inhibiting Casp-1 and knocking down NLRP3 led to the inhibition of SVA-induced IL-1β production, which demonstrated that SVA induced IL-1β production by regulating the NLRP3 inflammasome.

Our research first identified the mechanisms of NLRP3 activation by SVA infection. As reported, RNA induces NF-κB activation through PRR recognition, which acts as “signal 1” to induce the pro-IL-1β and NLRP3 transcription (24). We found that SVA RNA can induce IL-1β production. SVA has a +ssRNA genome, and it can recognize through RIG-I or MDA5 to induce NF-κB phosphorylation (38). Therefore, SVA genomic RNA may act as a “fuse” of “signal 1.” Similar to HCV, the 3'UTR of RNA genome contained the crucial motif for IL-1β induction (41), we speculated that the SVA RNA genome also might have the specific sequence or critical structure for SVA RNA-induced IL-1β secretion. The mechanism needs to be revealed further. Inhibiting RNA translation changed IL-1β secretion into a normal resting state. SVA encodes four structural proteins and eight nonstructural proteins. In this study, we showed that the SVA 3D protein is required for NLRP3 inflammasome activation, which has not been reported previously. We also found that 3D also can induce NF-κB activation. After 3D expression, NF-κB promoter activation and phosphorylation were higher than control. Meanwhile, we found that 3D interacts with IKKα and IKKβ to induce NF-κB activation and nucleus translocation. These results suggested that the effects of SVA RNA and 3D induction of NF-κB activation are superimposed. The initiation of “signal 1” results in nuclear factor-kappa B (NF-κB) activation and leads to the transcription of pro-IL-1β and NLRP3.

The SVA 3D protein is known as a viral polymerase that synthesizes the viral genome. Here, we reveal a new function of SVA 3D in NLRP3 inflammasome activation. “Signal 2” is activated by three models to induce NLRP3 inflammasome assembly, ultimately forming the activation of caspase-1 and IL-1β. The 3D protein interacts with NLRP3 through the NACHT domain using its 1 to 154 aa fragment to promote NLRP3-ASC speck structural formation. While 3D is localized in both the cytoplasm and nucleus, NLRP3 is diffusely distributed in the cytosol. 3D is colocalized with NLRP3.

A previous study showed that NLRP3 inflammasome activation requires the induction of “signal 2” (42). There are three models for “signal 2” induction. The ROS model, which invigorates the circulation of K^+^ and induces NLRP3 inflammasome activation (28, 43), the lysosomal rupture model, which causes the release of cathepsin B after lysosomal damage, leads to NLRP3 activation (44, 45), and the ion channel model, which regulates the concentration of K^+^ or Ca^2+^ in the cells, ultimately helps pathogen-associated molecular patterns (PAMPs) and damage-associated molecular patterns (DAMPs) to enter into the cytosol or cause mitochondrial dysfunction to activate the NLRP3 inflammasome. We found that ROS production and the lysosomal rupture model are not factors for NLRP3 inflammasome activation in SVA infection and 3D expression but depended on the ion channel model. We found that SVA infection and 3D expression decreased IL-1β secretion after treatment with BAPTA-AM at different gradients. In addition, using a high concentration of Ca^2+^ facilitates IL-1β secretion, which was induced by SVA infection or 3D expression but reversed the results with K^+^. At present, how 3D leads to ion concentration changes and the relationship between 3D-NLRP3 interactions and ion concentration changes is still unclear. As in many Ca^2+^ signal transduction pathways, Ca^2+^ is mobilized from extracellular and intracellular stores (46). The endoplasmic reticulum (ER) is the main store of intracellular Ca^2+^, and ER Ca^2+^ release is essential for inflammation (47). The Ca^2+^ release channel of IP3R in the ER is related to NLRP3 inflammasome activation by many stimuli, and many NLRP3 inflammasome stimuli may critically promote ER Ca^2+^ release through IP3R or other Ca^2+^ channels (48, 49). Therefore, it is reasonable to speculate that the relationship between 3D-NLRP3 binding and ion concentration changes may be related to organelle stress, such as ER stress. The exact mechanisms need to be further studied.

Most importantly, the biological effects of SVA infection on the induction of IL-1β responses were evaluated in mice and natural hosts, and the results showed that the IL-1β mRNA level and protein production in the heart, kidney, liver, and spleen became stronger in 3D-expressing pigs. Meanwhile, 3D protein expression induced the mice organs congestion, swelling, and inflammation.

**FIG 9:**
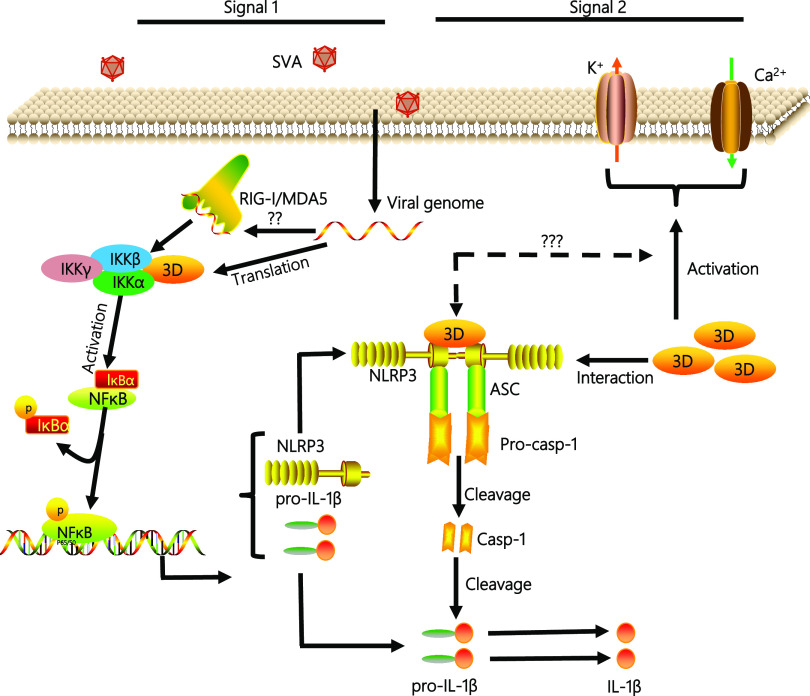
A model of the 3D mechanism by which SVA induces NLRP3-mediated inflammatory responses. Upon SVA infection, first, the SVA RNA genome is recognized by RIG-I-like receptors and NF-κB-induced pro-inflammatory cytokine production. SVA 3D protein also induces NF-κB activation to increase the NLRP3 and pro-IL-1β transcription during SVA infection. Second, 3D using its N-terminus (aa 1 to 154) interacts with the NATCH domain of NLRP3 to facilitate the assembly of the 3D-NLRP3-ASC complex to induce the pro-Casp-1clevaged into Casp-1, which caused IL-1β to change from a precursor form to a mature form. Moreover, 3D induces cytosolic K+ efflux and Ca^2+^ influx to promote NLRP3 activation. Taken together, 3D leads to the activation of the NLRP3, ultimately causing IL-1β production and secretion. Then, IL-1β is involved in the initiation of pro-inflammatory signal transduction during the SVA infection.

In conclusion, as shown in Fig. 9, the mechanism by which SVA infection induces the NLRP3-mediated inflammasome to promote IL-1β production was uncovered. In this study, we found two ways of IL-1β secretion induced by SVA infection: (i) the SVA genome can induce the production of IL-1β. We speculate that the SVA genome can be recognized by the RIG-I-like receptor of RIG-I/MDA5 and then induce the activation of NF-κB, which leads to the upregulation of NLRP3 and pro-IL-1β transcription. However, further work needs to reveal how the SVA genome promotes NLRP3 inflammatory complex assembly. (ii) SVA 3D gene transcription and translation to form 3D protein. 3D protein promotes the activation of NF-κB by binding IKKα and IKKβ, which upregulates the NLRP3 and pro-IL-1β transcription. Then, the N-terminal of 3D promotes the assembly of the NLRP3 inflammatory complex to induce IL-1β production by binding to the NACHT domain of NLRP3. At the same time, 3D protein also affects the production of IL-1β through ion channels. The relationship between the effect of 3D on ion channels and the 3D-NLRP3 complex formation also needs further study.

## MATERIALS AND METHODS

### Animals, clinical specimens, and blood samples.

Sera of SVA-infected pigs (*n* = 9) and healthy pigs (*n* = 9) were collected from the Animal Biosafety Level-3 (ABSL-3) lab of the Lanzhou Veterinary Research Institute (Lanzhou, Gansu, China). Male C57BL/6, 60-day-old mice were bought from an animal production house (Lanzhou veterinary research institute). Pig bone marrow-derived macrophages (BMDMs) were collected from 3-month-old pigs. BMDMs were cultured in RPMI 1640 in the presence of granulocyte-macrophage colony-stimulating factor with 10% heat-inactivated FBS for 5 days. The culture medium was replaced every other day. Pigs (3 months) and 60-day-old C57BL/6 mice were mock infected with DMEM or infected with empty lentivirus or 3D expression lentivirus (1 × 10^8^ TU/mL) by intramuscular injection for 7 days. Tissues collected from pigs were used for cytokines detection. According to the Declaration of World Medical Assembly (WMA), the animal study was directed and approved by the Lanzhou Veterinary Research Institute (NO. LVRIAEC-2021-012). Animal ethics were strictly followed during the animal study.

### Cells lines and cultures.

Porcine kidney (PK-15) (ATCC#CCL-33), human embryonic kidney 293 (HEK293) (ATCC #CRL-1573), human monocytic cell line (THP-1) (ATCC #TIB-202), and Instituto Biologico-Rim Suino-2 (IBRS-2) (ATCC #CRL-1835) cells were purchased from the American Type Culture Collection (USA). PK-15 and IBRS-2 cells were cultured in minimum essential medium (MEM, Gibco, USA) supplemented with 10% heat-inactivated FBS, penicillin (100 U/mL), and streptomycin sulfate (100 μg/mL). Human embryonic kidney 293 (HEK293) cells were cultured in DMEM (Gibco, USA) supplemented with 10% heat-inactivated FBS, penicillin (100 U/mL), and streptomycin sulfate (100 μg/mL). The THP-1 human monocytic cell line was cultured in RPMI 1640 (Gibco, USA) supplemented with 10% heat-inactivated FBS, penicillin (100 U/mL), and streptomycin sulfate (100 μg/mL). All cells were maintained at 37°C with 5% CO_2_.

### Viruses.

Strain of SVA was isolated from Fujian, China, and maintained by the National Foot and Mouth Diseases Reference Laboratory, Lanzhou Veterinary Research Institute, Chinese Academy of Agricultural Sciences. SVA was used for viral challenge and propagated in IBRS-2 cells. Sendai virus (SeV) was maintained by our laboratory.

### Reagents and antibodies.

Nigericin (CAS number 28643-80-3) and Caspase-1 inhibitor VX-765 (Cat# inh-vx765i-5) were purchased from the InvivoGen Biotech Co., Ltd. (USA). The monoclonal antibodies mouse anti-FLAG (Cat#8146), mouse anti-Myc (Cat#2276), mouse anti-β-actin (Cat#3700), mouse anti-HA (Cat#2367), rabbit anti-IL-1β (Cat#12703), rabbit anti-NLRP3 (Cat#15101), rabbit anti-Casp-1 (Cat#24232), and rabbit anti-ASC (Cat#67824) were purchased from Cell Signaling Technology (USA). Rabbit polyclonal SVA 3D antibody was prepared by us using purified SVA 3D protein. MCC950 (Cat# HY-12815A) (NLRP3 activation inhibitor) was purchased from MCE (Shanghai, China). The translation inhibitor CHX (Cat# A8244) was purchased from APEXBIO (Houston, TX, USA). Lipofectamine 2000 (Cat# 11668019) was purchased from the Thermo Fisher Scientific, USA.

### Plasmids construction.

A myc-tagged NLRP3 and Casp-1 plasmid were generated by inserting full-length NLRP3 and Casp-1 cDNA fragments into pcDNA3.1/Myc A vector (Invitrogen, Carlsbad, CA, USA). Genes encoding viral structural and non-structural proteins were amplified from the SVA genome and cloned into the Flag-CMV-7.1 vector (Sigma-Aldrich, USA) to construct plasmids expressing Flag-tagged viral proteins using standard molecular biology techniques. Flag-tagged 3D mutants (Δ1 to 154; Δ154 to 309; and Δ309 to 463) and myc-tagged NLRP3 mutants (PYD, NACHT, and LRR) were constructed as described above. All constructed plasmids were analyzed and verified by DNA sequencing.

### Lentivirus production and infection.

The target sequence of NLRP3 shRNA was

(i) 5^/^-AGAGAAGGCAGACCATGTGGATCTAGCCA-3′^/^,

(ii) 5^/^-CAGTCTGATTCAGGAGAACGAGGTCCTCT-3′^/^,

(iii) 5^/^-GAGACATTCTCCTGAGCAGCCTCATCAGA-3′^/^,

(iv) 5^/^- GTACGTGAGAAGCAGATTCCAGTGCATTG-3′^/^.

Annealed shRNA synthesized cDNA fragments corresponding to the cDNAs of NLRP3 genes were digested with BamHI and EcoRI and ligated into pLVX vector (HANBIO, Shanghai, China), named as lentivirus expressing NLRP3 shRNA (shNLRP3). Negative control (sh-NC), an ineffective shRNA cassette in the pGFP-C-shLenti shRNA vector plasmid, was purchased from ORIGENE (Rockville, USA). SVA 3D was digested with BamHI and EcoRI and ligated in the LV011-pHBLV-CMV-MCS-3flag-EF1-T2A-Zsgreen-Puro (HANBIO, Shanghai, China). The Lentivector encoding shNLRP3 and Lenti-3D were transfected into HEK293T cells together with psPAX2 and pMD2.G with Lipofectamine 2000. The primers are shown in Table S1. Culture supernatants were harvested at 48 h and 72 h. Supernatants were collected at 48 h and 72 h, filtered with a 0.45 μm filter, and after that centrifuged at 72,000 × *g* for 120 min at 4°C. BMDM cells, PK-15 cells and pigs were infected with the supernatants containing lentiviral particles. shRNA knockdown efficiencies were assessed by Western blot analysis. The IL-1β level and IL-1β mRNA levels from infected cells and infected pig organs were measured by ELISA and qRT-PCR.

### Coimmunoprecipitation assay.

PK-15 cells were cultured in 10 cm^2^ plates. The cells were transfected by multiple plasmids (SVA 3D and its mutants, NLRP3, NACHT, LRR, PYD, NF-κB, IKKα, IKKβ, IKKγ). After 24 h, cells were collected and lysed in 0.8 mL of lysis buffer (20 mM Tris (pH 7.5), 150 mM NaCl, 1% Triton X-100, 1 mM EDTA, 10 mg/mL aprotinin, 10 mg/mL leupeptin, and 1 mM PMSF). Lysates were incubated with 0.3 μL of anti-Flag, anti-Myc, anti-HA antibody, or control IgG and 50 μL of G-Sepharose (GE Healthcare) for 6 h to8 h. The Sepharose beads were washed three times with 1 mL of lysis buffer containing 500 mM NaCl. Immunoblotting was used to analyze precipitates.

### Confocal microscopy.

Nunc glass-bottom dishes were used to culture PK-15 cells. After 24 h of transfection with Lipofectamine 2000, the cells were fixed with 4% paraformaldehyde for 30 min. After that, the cells were permeabilized with 0.1% Triton X-100 for 15 min. Then, the cells were incubated in 5% BSA for 1 h at RT. The cells were incubated with primary antibody overnight and secondary antibody (Alexa Fluor 488- or 594-conjugated secondary antibody) for 1 h. The images were acquired with a laser-scanning confocal microscope (LSCM, Leica SP8, Solms, Germany). For virus infection assays, Porcine BMDM cells were infected with SVA at a MOI of 4 at 37°C for 16 h. The SVA-infected or mock-treated cells were operated as described above.

### Western blot analysis.

The target proteins were resolved by sodium dodecyl sulphate–polyacrylamide gel electrophoresis (SDS-PAGE) and transferred to an Immobilon-P membrane (Millipore, USA) for Western blotting. The membrane was blocked in 5% skim milk and incubated with sufficient primary and secondary antibodies. Enhanced reacting chemiluminescence (Thermo Fischer, USA) was used to visualize antibody-antigen complexes.

### RNA extraction and RT-PCR.

Total RNA was extracted using TRIzol Reagent (Invitrogen, USA). M-MLV reverse transcriptase (Promega, USA) and random hexamer primers (TaKaRa, Japan) were used to prepare the cDNA. The generated cDNA was used as a template for SVA RNA and cellular mRNA host expression. Real-time PCR (RT-PCR) to measure the abundance of different mRNAs was conducted using Mx3005P qPCR (Agilent Technologies, USA) and SYBR Premix *Ex Taq* reagents (TaKaRa, Japan). The data were normalized to GAPDH expression. The 2^−ΔΔCt^ method was used to calculate the relative expression of mRNA.

### Luciferase reporter assays.

PK-15 cells (1 × 10^5^) were seeded in 48-well plates and transfected with 100 ng of NF-κB reporter plasmid, 20 ng of pRL-TK, and 200 ng of the SVA 3D plasmids, (NF-κB and pRL-TK reporter plasmids are gifts kindly from Shu Hong Bing’s Lab, Wuhan University, China). After 24 h hpi, cells were then mock-infected/treated or infected/treated with SeV/SVA for 16 h. According to the manufacturer’s protocol, the luciferase activity was measured using the Dual-Luciferase Reporter Assay System (Promega). The data represent relative firefly luciferase activity normalized to Renilla luciferase activity. Cell lysates were further used in Western blotting to evaluate protein expression.

### ASC oligomerization.

The cell lysate supernatants were combined with SDS charging buffer to evaluate Western blots using an ASC antibody. Three washes were conducted on the pellets with phosphate-buffered saline (PBS), and after 30 min, they were connected with fresh disuccinimidyl suberate (DSS) (2 mM, Sigma). The related pellets were centrifuged and mixed for Western blot analysis using SDS loading buffers.

### Enzyme-linked immunosorbent assay.

To detect the level of IL-1β in sera, organs, and supernatants of the cultured medium, enzyme-linked immunosorbent assay (ELISA) kits (human, pigs) were purchased from RayBiotech (USA).

### Mature IL-1β measurement.

The supernatant (1 mL) of cultured cells was collected in cryogenic vials (Corning) and stored frozen at −80°C for 24 h. A rotational vacuum concentrator (Christ-Alpha 1-2LDplus) was used to lyophilize the samples. The lyophilized product was dissolved in 100 μL PBS and mixed with SDS loading buffer for Western blot analyses using antibodies against mature IL-1β (Asp116 1:500; Cell Signaling).

### Statistical analysis.

All tests were reproducible, and similar findings were repeated at least three times. Using Tukey’s *post hoc* test, sample variation was determined and analyzed by one-way ANOVA. Means are represented with histograms, with error bars representing the mean (s.e.m.) standard error, and *P* values < 0.05 were considered statistically significant.
