# Supplementary material for: Senecavirus a 3D Interacts with NLRP3 to Induce IL-1β Production by Activating NF-κB and Ion Channel Signals
Source: Microbiol Spectr. 2022 Mar 7;10(2):e02097-21. doi: 10.1128/spectrum.02097-21 (PMC9045273; doi:10.1128/spectrum.02097-21)
Supplement: SUPPLEMENTAL FILE 1 — Supplemental material. Download SPECTRUM02097-21_Supp_1_seq11.pdf, PDF file, 1.1 MB [file spectrum02097-21_supp_1_seq11.pdf]

Supplemental Figure-1

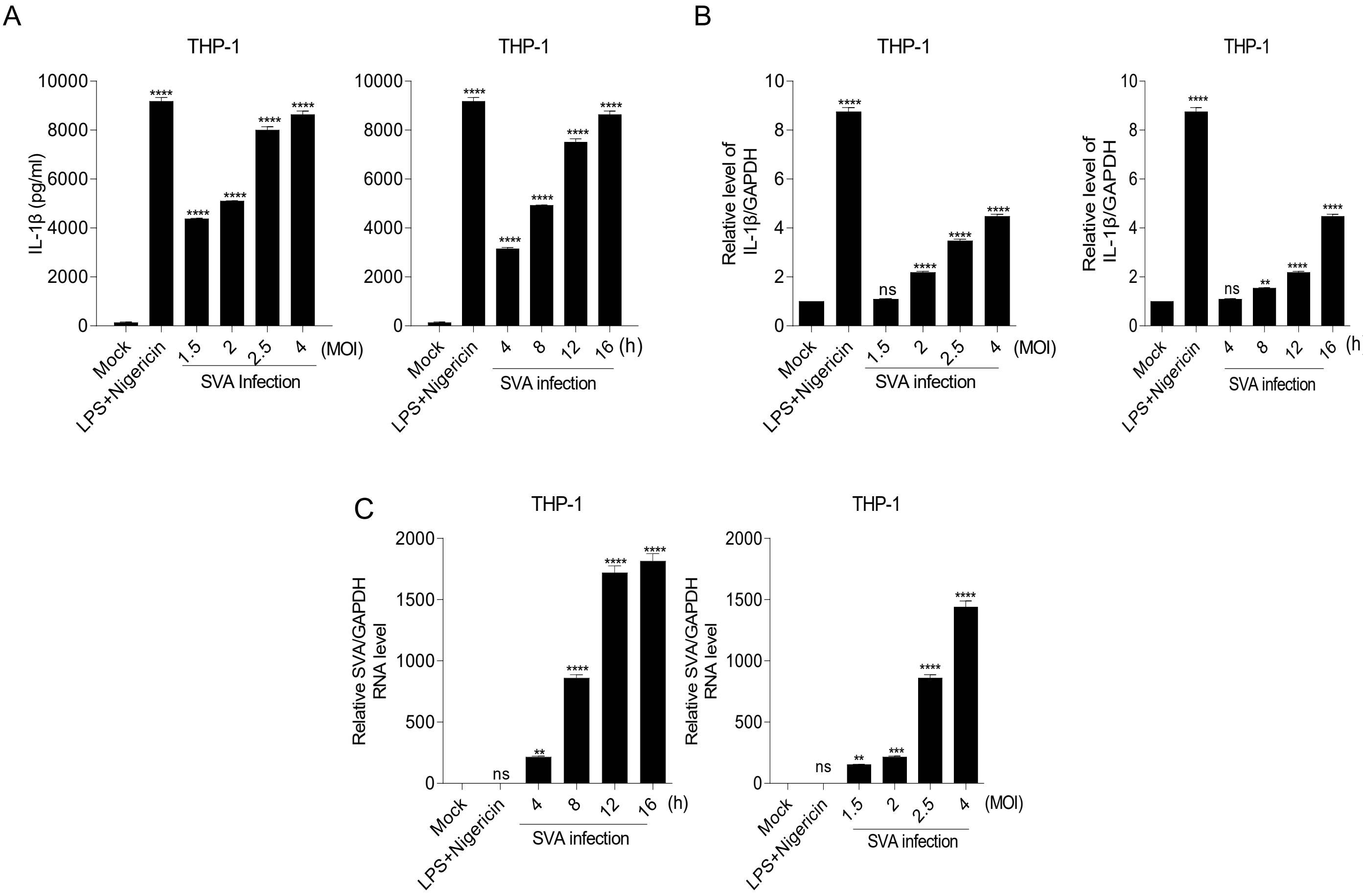

Supplemental Fig.1 SVA infection induces IL-1β secretion and production.

(A-C) THP-1 cells were treated with 2 μM Nigericin for 2 h pre-primed with LPS (60 ng/ml) for 8 h or infected with SVA for 16 h at MOI= 1.5, 2, 2.5, 4, or at MOI=4 for 4, 8, 12, 16 h. The IL-1β levels in the medium were detected by ELISA **(A)**. The IL-1β mRNA levels at the indicated times and MOIs were determined by qPCR **(B).** The SVA mRNA levels at the indicated times and MOIs were determined by qPCR **(C)**

Supplemental Figure-2

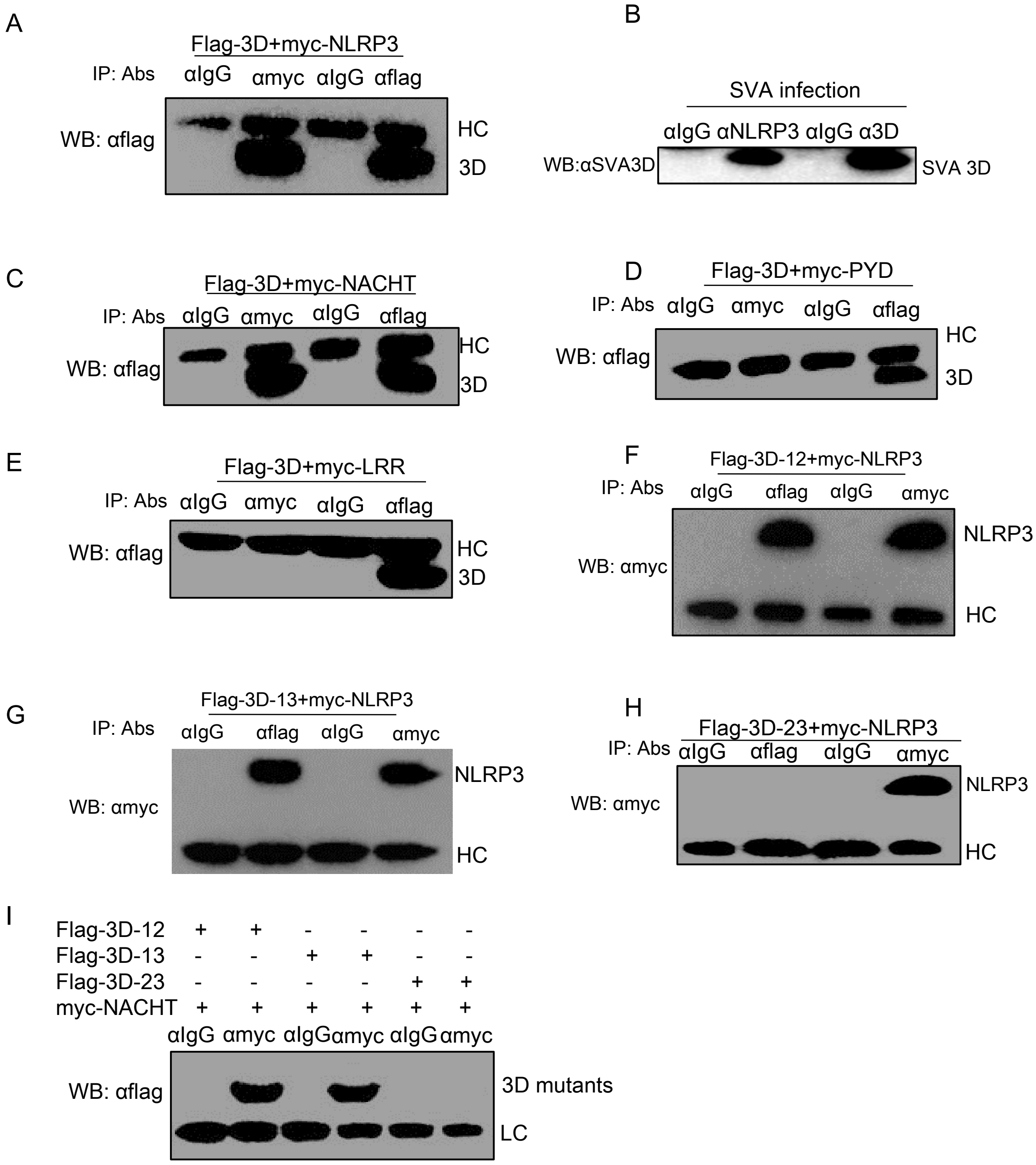

Supplemental Fig.2 The SVA 3D N-terminus (1-154 amino acids) binds with the NACHT domain of NLRP3 to facilitate NLRP3 inflammasome assembly and activation.

(A, C-E) PK-15 cells were transfected with 5 µg Flag-3D along with the 5 µg myc-NLRP3, myc-NACHT, myc-PYD, or myc-LRR- the domain of NLRP3. Cell lysates were subjected to IP using IgG, anti-myc, or anti-Flag primary antibody and detected with western blotting using the indicated antibody (WB). **(B)** PK-15 cells were infected with SVA. Cell lysates were subjected to IP using IgG, anti-NLRP3, or anti-SVA 3D primary antibody and detected with western blotting using the SVA 3D polyclonal antibody (WB). **(F-I)** 5 µg of the SVA 3D deletion mutants (Flag-3D-13, Flag-3D-12, Flag-3D-23) co-expressed with 5 µg myc-NLRP3 or myc-NACHT. After 24 h, protein samples were subjected to IP with the indicated antibody and detected by western blotting. After transfection, samples were harvested for 24 hour. For western blot, the antibody dilution ratio was 1: 1000.

Supplemental Figure- 3

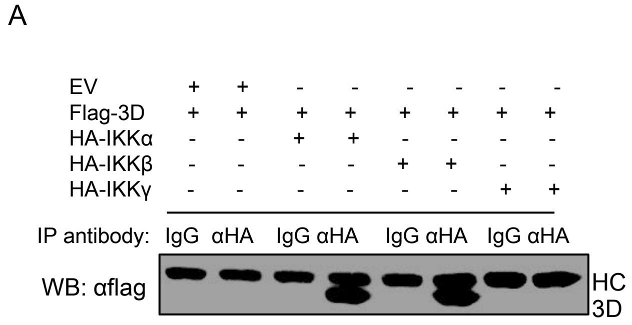

Supplemental Fig.3 SVA 3D -induced NLRP3 activation depends on the NF-κB pathway

(A) PK-15 cells were transfected with 5 μg Flag-3D along with the 5 μg HA-IKKα, HA-IKKβ, and HA-IKKγ. Cell lysates were subjected to IP using IgG, anti-HA, or anti-Flag primary antibody and detected with western blotting using the indicated antibody (WB).

Supplemental Figure-4

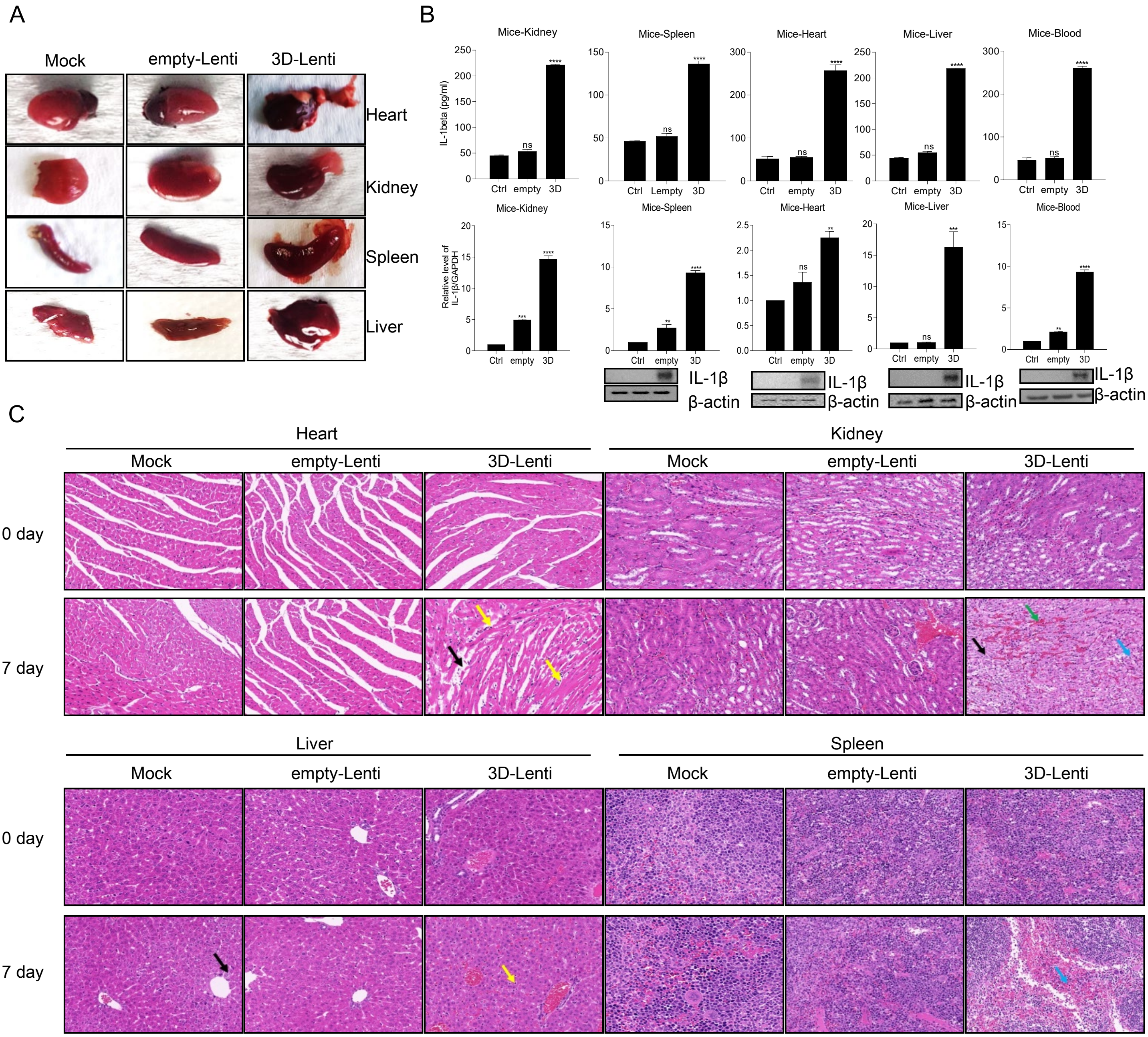

Supplemental Fig.4 SVA 3D induced an inflammatory response in animals.

(A-C) C57BL/6 mice (60 days old) were treated with DMEM or infected with empty lentivirus (1×10^7 TU/ml) or 3D expression lentivirus (1×10^7TU/ml) (each of them n=3) by tail vein injection for 7 days. The phenotypic changes and histopathological changes (HE staining) of organs were observed **(A, C)**. The levels of IL-1β of the mice organs (blood, liver, kidney, spleen, and heart) were determined by ELISA, qPCR, and western blotting **(B).**

Table 1: Primers used in this study for the RT-PCR

Genes	Sense primers (5/-3/)	Anti-sense primers (5/-3/)
hGAPDH-qRT	GAGTCAACGGATTTGGTCGT	GACAAGCTTCCCGTTCTCAG
P-GAPDH-qRT	ACATGGCCTCCAAGGAGTAAGA	GATCGAGTTGGGGCTGTGACT
mGAPDH-qRT	CCATGTTCGTCATGGGTGTGAACCA	GCCAGTAGAGGCAGGGATGATGTTC
h-p65-qRT	TGAACCGAAACTCTGGCAGCTG	CATCAGCTTGCGAAAAGGAGCC
h-IL-1β-qRT	GCAAGGGCTTCAGGCAGGCCGCG	GGTCATTCTCCTGGAAGGTCTGTGGGC
P-IL-1β-qRT	GACGGGCTTTTGTTCTGCTT	GGACATGGAGAAGCGATTTGT
SVA- qRT	AGAATTTGGAAGCCATGCTCT	GAGCCAACATAGARACAGATTGC
m-IL-1β-qRT	GCACTACAGGCTCCGAGATGAAC	TTGTCGTTGCTTGGTTCTCCTTGT
P-caspase 1-qRT	GAAATACTCCGAAGAAGTCCCAGA	GACCCCTTGCTTCTCACCAC
P-PYCARD-qRT	TCAAGGGTCACAGACGTGGA	TTTGGTGGGGTTGGTGTG
h-NLRP3-qRT	AAGGGCCATGGACTATTTCC	GACTCCACCCGATGACAGTT
h-ASC-qRT	AACCCAAGCAAGATGCGGAAG	5’-TTAGGGCCTGGAGGAGCAAG-3’
h-Casp-1-qRT	TCCAATAATGCAAGTCAAGCC	GCTGTACCCCAGATTTTGTAGCA
SVA-3D- qRT	AGAATTTGGAAGCCATGCTCT	GAGCCAACATAGARACAGATTGC

P :porcine, h: human, m:mouse
